# FUNCTIONAL AND CELLULAR EVALUATION OF THE LIVER AFTER LOW-POWER LASER STIMULATION DURING SURGERY

**DOI:** 10.1590/0102-6720201700020010

**Published:** 2017

**Authors:** Yasna Patrícia Aguilera GODOY, Simone GERSON, Milene Santana PINTO, Márcio Fernando BOFF, Marcello Ávila MASCARENHAS, Valesca Veiga CARDOSO

**Affiliations:** 1 Methodist University Center - IPA, Laboratory of Toxicology and Mutagenesis, Porto Alegre, RS;; 2 Oncologic Surgery Service, Mãe de Deus Hospital, Porto Alegre, RS, Brazil.

**Keywords:** Mutagenesis, Hepatectomy, Liver regeneration, Laser therapy

## Abstract

**Background:**

Partial hepatectomy is a surgical intervention of the liver that can trigger its regenerative process, where the residual lobes deflagrate a compensatory hyperplasia, causing its restoration almost to the original volume. Nevertheless, depending on the extent of liver damage its regeneration might be impaired. The low-power laser has been studied with beneficial results.

**Aim:**

To investigate the possible functional and mutagenic damage arising from the use of low-power laser used in liver regeneration after partial hepatectomy.

**Methods:**

Fifteen male adult Wistar rats were hepatectomizated in 70% and laser irradiated or not with dose of 70 J/cm^2^, 650 nm, 100 mW, directly on the remaining liver, during the perioperative period. These animals were divided into four groups: G1 (control, 7 days); G2 (laser, 7 days); G3 (control, 14 days); G4 (laser, 14 days). Were analyzed the liver weight; number of hepatocytes; deposition of collagen fibers; liver function tests: serum alanine aminotransferase, aspartate aminotransferase, alkaline phosphatase, gamma glutamyl transferase, bilirubin and micronucleus test in peripheral blood erythrocyte.

**Results:**

The liver weight was greater in G3 and G4 (p=0.001 and p=0.002) compared to other groups. The deposition of collagen fibers in G1 was statistically higher than the other groups (p=0.01). In tests of liver function and micronucleus test was not found significant differences between the studied groups.

**Conclusion:**

Low-power laser stimulation did not cause loss of liver function or mutagenic damage.

## RESUMO

**Racional:**

A hepatectomia parcial é intervenção cirúrgica que pode desencadear processo regenerativo, onde os lobos residuais deflagram resposta de hiperplasia compensatória, ocasionando restauração próxima ao seu volume original. Contudo, dependendo da extensão das lesões hepáticas a regeneração pode ser prejudicada. O laser de baixa potência tem sido pesquisado com resultados benéficos no processo de regeneração hepática.

**Objetivo:**

Investigar os possíveis danos funcionais e mutagênicos decorrentes da utilização do laser de baixa potência utilizado na regeneração hepática após hepatectomia parcial**.**

**Métodos:**

Quinze ratos adultos Wistar foram hepatectomizados a 70%, irradiados ou não com laser, dose de 70 J/cm^2^, 650 nm,100 mW, de forma direta sobre o fígado remanescente, durante o período transoperatório. Os animais foram distribuídos em quatro grupos: G1 (controle, 7 dias); G2 (laser, 7 dias); G3 (controle 14 dias); G4 (laser,14 dias). Foram analisados o peso do fígado; número de hepatócitos; deposição de fibras colágenas; teste de função hepática: alanina aminotransferase, aspartato aminotransferase, fosfatase alcalina, gama glutamiltransferase, bilirrubinas e teste de micronúcleo em eritrócitos.

**Resultados:**

O peso do fígado apresentou-se aumentado nos grupos G3 e G4 (p=0,001 e p=0,002) comparados aos demais grupos. A deposição das fibras colágenas no G1 foi estatisticamente maior em relação aos demais grupos (p=0,01). Nos testes de função hepática e teste de micronúcleo não foram encontradas diferenças significativas entre os grupos.

**Conclusão:**

O laser de baixa potência não ocasionou perda de função hepática ou dano mutagênico.

## INTRODUCTION

Partial hepatectomy (HP) is a simple technique consisting of the surgical removal of the two largest lobes of the liver, median and left lateral, corresponding in approximately 67% two thirds of the original hepatic mass^10^. This surgical procedure can initiate the regenerative process, where the residual lobes deflate compensatory hyperplasia response, restoring the liver to its original volume^**11,15,23, A, B, C**^ **.**

The surgical technique preserves in the residual tissue 1/3 of the hepatocytes^15^, which are cells that have great proliferative potential; they are resting in normal livers and replicate in a limited and regulated way in hepatic regeneration, stimulated by HP^6^. The kinetics of cell proliferation and the growth factors produced by hepatocyte proliferation suggest that these cells provide myogenic stimuli that lead to the proliferation of other cells, starting in the DNA synthesis of hepatocytes^15^.

Collagen is a protein derived from the extracellular matrix, and can perform various functions in the body^14^. In the liver, when an injury occurs, at the first moment there is irregular deposition of collagen, which tends to decrease as the liver tissue regenerates^27^. However, when there is a very extensive and permanent lesion, the process of reducing collagen excess does not occur and there is no architectural division, forming fibrotic areas that can be stimulated by infectious processes or by overexposure of the liver to potentially irreversible toxic substances, considered cirrhosis .

The ability of the liver to regenerate is crucial after HP, since massive loss of hepatocytes, as well as cell necrosis or apoptosis, may be limiting factors for cell replication and consequently delayed hepatic regeneration process, which can cause serious adverse effects on animal survival^12^.

Low-power or low intensity laser therapy (TLBP) uses photons with wavelengths located in the visible red region of the spectrum, or in the near infrared region, and consist of monochromatic, relatively low-power diode lasers (below 500 mW) and are used in athermic treatments, since they do not cause heat in biological tissues^13^.

The effect of TLBP on hepatic regeneration after HP stimulates a significant improvement of hepatic regeneration, favorable for the formation of new hepatocytes, mesenchymal stem cells and angiogenesis in regeneration in rats^22^, improving the stability of mitochondrial function, increasing the rate of respiratory control, suggesting delay of hepatocellular overload of the remaining liver^4^ ^,^ ^7^. TLBP can lead to good clinical performance, simple, fast and easy to perform, promoting an improvement in the regenerative capacity of the organ after HP, by increasing the expression of the hepatocyte growth factor, exerting an accelerating role in the process of liver regeneration in rats, regardless of the penetration depth of the laser^3^ ^,^ ^20^.

The evaluation of liver function is based on liver function tests, through sensitive and specific markers, which are classified according to hepatocellular activity, where the diagnosis results in increased values ​​in the blood stream^8^ ^,^ ^18^. In hepatocyte membrane lesions, elevated serum levels of the enzymes aspartate aminotransferase (AST) and alanine aminotransferase (ALT) are found; cholestatic diseases, due to the impediment or reduction of bile flow, lead to an increase in plasma levels of bilirubin and predominantly alkaline phosphatase (AF) and gamma glutamyl transpeptidase (GGT). Their plasma increases result from infiltrating cholangiocyte lesions in the increase of AF, GGT and, occasionally, bilirubin^14^ ^,^ ^18^.

Studies that observe liver function through liver function tests, through analysis of serum levels of aminotransferases, gamma glutamyltransferase, alkaline phosphatase and bilirubin, show that the use of TLBP in the hepatic regeneration process does not cause liver damage in addition to the surgical procedure on livers of healthy rats^2^ ^,^ ^17^ ^,^ ^20^ ^,^ ^21^.

In the degradation of collagen fibers in the hepatic tissue remaining after HP, TLBP avoids the degradation of fibrotic areas and subsequent continuity of cellular disarrangement^17^ ^,^ ^21^.

The micronucleus test is used as a biomarker in the detection and monitoring of diseases associated with chromosomal mutation^5^, in which the micronucleus assay model can be used in peripheral blood erythrocytes^9^. Micronucleus is an accessory nucleus, originated from the breakdown of chromosomes, which are not included in the main nucleus during the telophase of mitosis or meiosis, remaining immersed in the cytoplasm^24^. Their presence suggests spontaneous genetic alterations or by induction through genotoxic agents^5^.

 The use of the low-power laser has been studied in the last decades as a therapeutic agent in the regeneration process of the hepatic tissue, after HP. The literature has reported several positive findings due to its use; however, there are deficiencies in the publications on the evaluation of cellular damage as an adverse effect resulting from its use.

 The objective of this study was to investigate the possible biological damages caused by the use of the low-power laser used in hepatic regeneration after partial hepatectomy.

## METHODS

All protocols followed the standards of the Brazilian College of Animal Experimentation (COBEA), based on the National Guide for the Care and Use of Laboratory Animals (National Research Council). This project was approved by the Ethics Committee on the Use of Animals (CEUA) of the Centro Universitário Metodista IPA n° 003/2014. It is a quantitative, comparative experimental study carried out at the Methodist University Center - IPA, Porto Alegre, RS, Brazil, in Toxicology laboratories, Mutagenesis in the period from April 2014 to June 2014.

The research population consisted of 15 Wistar rats, males, adults (90 days) weighing around 250-300g, kept in the Bioterio of the Centro Universitário Metodista. The animals were kept in plastic boxes, containing wood shavings, identified with labels according to the group, grouped into two animals per box. For identification, markings were used on the tail, performed with a permanent pen. The rats were kept under the conditions of the vivarium, with light controlled in a cycle of 12 h (dark light), temperature of 22-24° C, relative humidity of 70.5%. Feed and water were given ad libitum.

All rats had 70% HP (removal of the median and left lateral lobes)^12^, with or without TLBP irradiation directly in the remaining liver tissue during the transoperative period. The animals were randomly divided into four groups according to the euthanasia period: G1 (control, 7 days); G2 (laser, 7 days); G3 (control, 14 days); G4 (laser, 14 days). They were previously weighed and subsequently anesthetized with intraperitoneal injection of Ketamine 5% at a dose of 90 mg/kg, combined with 2% Xylazine at the dose of 12 mg/kg, producing sedation and effective surgical analgesia for 30 min. Their vital signs were monitored through an oximeter, in addition to the clinical evaluation of respiration through depth and rhythm, by the color of the membranes and mucous membranes, observing the effectiveness of pulmonary gas exchange. For maintenance of normothermia, the animals were kept warm with allogenic light (45W, 127V), with body temperature monitored by a digital rectal thermometer and maintained at around 37° C. The animals› foot reflex was evaluated every 5 min or more, in order to reach anesthetic depth. After the desired effect of the anesthesia the rats were submitted to tricotomy of the abdominal region. On sterile conditions, all were submitted to HP 70%. Then, before the closure of the abdominal cavity, the G2 and G4 groups received the TLBP of the diode laser beam, voltage 90-240 V, automatic, red laser emitter, indium-gallium-iodine-phosphorus (InGaIP) laser, 650 nm wavelength, maximum emitter power of 100 mW (Therapy XT model, brand DMC Equipamentos LTDA®, RDC 185/2001 (ANVISA) III, IEC 60825-1/3R) expanded to the entire remaining liver portion of each animal at a dose of 2 J, flow rate of 70 J/cm^2^, repeated at five different points, with 20 s application time per point. Experimental groups G1 and G3, considered as control groups, did not receive TLBP.

After these procedures, the rats had their abdominal cavities closed by means of simple suture. Once the surgical procedure was completed, they were kept warm with allogenic light and remained monitored until they were recovered from the anesthetic effects, when they were taken to the vivarium. To improve the survival rate in the immediate postoperative period, 20% glucose was conditioned in water^29^.

After the 7-14 days of the surgical procedure, according to the observation period of each group, the animals were euthanized for the dissection of the samples. Soon after euthanasia, livers were immediately removed for analysis of hepatocyte function, and blood samples were collected and centrifuged. Serology was used for biochemical analysis of liver function assessment using ALT, AST, AF, GGT and bilirubin. Hepatic function was observed through kinetic methods using commercial kits (Labtest®). The aminotranferases (ALT and AST) were serum-dosed by the UV-IFCC kinetic method; GGT and FA by modified Szasz; bilirubins by colorimetric method (Sims-Horn). All dosages were performed in triplicates in the Labquest semiautomatic system (Labtest®).

Hepatic tissue was weighed, cut into slides and fixed with 10% formaldehyde for 24 h for further processing and histological analyzes. For histology the livers were weighed, sectioned and processed conventionally, that is, after fixation with formalin, the sections were washed in distilled water, dehydrated in increasing series of ethanol, diaphanized and embedded in paraffin. The samples were sectioned in a microtome at a thickness of 7 μm and placed on glass slides identified with the group code and the animal number. The paraffin was removed from the cuts and the tissue hydrated, followed by H&E staining and Masson’s trichrome. The histological sections were analyzed and documented in a binocular biological microscope with Tim-2 Opton image capture. Healing areas and hepatic parenchyma morphology were evaluated in H&E stained sections to assess whether there was stimulation of remaining hepatic tissue hyperplasia after TLBP by counting the number of hepatocytes in 10 fields for each cut, increasing by 400x. The area analyzed corresponded to 62500 μm. For the sections stained in Masson trichrome, the proliferation of collagen fibers and reepithelialization were analyzed in 10 fields with a magnification of 200x per slide and assigned three scores: 0 (absent), 1 (light), 2 (moderate) and 3 (sharp) .

The evaluation of the mutagenic damage occurred through the micronucleus count in peripheral blood erythrocytes. After euthanasia blood samples were immediately placed on a slide and then stained with Giemsa®. They were analyzed by optical microscopy, using an immersion objective magnification (400x), being determined from a thousand erythrocytes per slide. The classification and micronucleus count in erythrocytes followed the protocol suggested by Tolber et al^28^.

### Statistical analysis

 The results were expressed as mean and standard deviation, using one-way ANOVA, followed by the Tukey test as post-hoc, and expressed as mean±standard deviation. The value used for statistical significance was p<0.05. The database and analyzes were performed in the Statistical Package for the Social Sciences (SPSS version 21.0 for Windows).

## RESULTS

Table 1 shows the comparison of liver weight between groups by the mean±standard deviation: G1 7 day control, 8.13±0.10 g; G2 laser 7 days, 8.53±0.20 g; G3 control 14 days, 10.04±0.03 g; G4 laser 14 days, 10.10±0.06 g. The results showed that in the G3 group (p=001), livers weights were significantly higher than in G1 and G2. Likewise, it in G4 (p=0.02) was significantly higher than in G1 and G2. The comparison of the number of hepatocytes among the different groups was also observed: G1 control of 7 days, 555±17 U; G2 laser 7 days, 619±30 U; G3 control 14 days, 642±44 U; G4 laser 14 days, 714±5.6 U). The results did not present statistical significance among the four groups of the experiment in counting the number of hepatocytes. However, an increase in the number of hepatocytes, in the laser-treated groups compared with their respective controls could be observed - G2 vs. G1 and G4 vs. G3. 

**TABLE 1:** Comparison of hepatectomized liver weight and hepatocyte counts among different groups

Group	Weight (g) **	Number of hepatocytes ***
G1	8.13±0.10	555±17
G2	8.53±0.20	619±30
G3	10.04±0.03 * p=0.01	642±44
G4	10.10±0.06 * p=0.02	714±5.6

Values=mean standard deviation; one-way ANOVA and the Tukey post-test; * p<0.05

**FIGURE 1:**
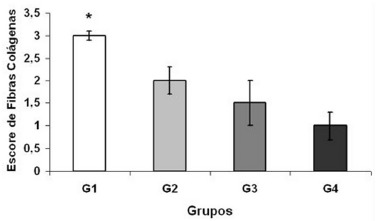
Quantification of collagen fibers

Figure 1 shows the quantification of collagen fibers in the livers between the different groups: G1 control 7 days, 3±0.1; G2 laser 7 days, 2±0.3; G3 control 14 days, 1.5±0.5 and G4 laser 14 days, 1±0.3. The results show that in group G1 the values referring to the presence of collagen fibers are significantly higher (p=0.01) in relation to the other groups.

0=absent; 1=mild; 2=moderate; 3=pronounced; values=mean and the standard deviation

**FIGURE 2:**
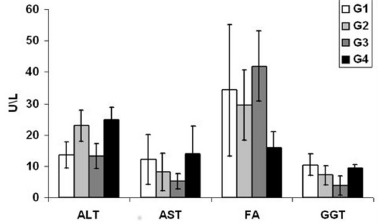
Comparison between ALT, AST, FA e GGT

Figure 2 shows the comparison of serum levels between the different groups of the enzymes. The ALT was in the G1 control 7 days, 13.5±4.2 U/l; G2 laser 7 days, 22.89±5 U/l; G3 control 14 days, 13.18±4 U/l; G4 laser 14 days, 24.67±4 U/l. The AST was in the control G1 7 days, 12.11±8 U/l; G2 laser 7 days, 8.22±6 U/l, G3 control 14 days, 5.19±2.45 U/l; G4 laser 14 days, 14±8.71 U/l. AF was in G1 control 7 days, 34.21±21 U/l; G2 laser 7 days, 29.49±11.1 U/l; G3 control 14 days, 41.92±11.28 U/l; G4 laser 14 days, 16±5 U/l. The GGT was in the G1 control 7 days, 10.44±3.5 U/l; G2 laser 7 days, 7.0 ±3.07 U/l; G3 control 14 days, 3.87±3 U/l; G4 laser 14 days, 9.42±1.13 U/l.

Values=mean standard deviation; one-way ANOVA and the Tukey post-test; * p<0.05

**FIGURE 3:**
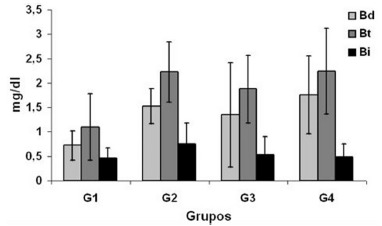
Comparison of total (Bt), direct (Bd) and indirect (Bi) bilirubin

Figure 3 shows the comparison of serum levels of direct, total and indirect bilirubin among the different groups, where it can be seen in the 7-day G1 control, 0.72±0.3 mg/dl; G2 laser 7 days, 1.53±0.36 mg/dl; G3 control 14 days, 1.35±1.07 mg/dl; G4 laser 14 days, 1.76±0.8 mg/dl. Total bilirubin was in group G1 control 7 days, 1.1±0.68 mg/dl; G2 laser 7 days, 2.23±0.62 mg/dl; G3 control 14 days, 1.88±0.69 mg/dl; G4 laser 14 days, 2.24±0.88 mg/dl. Indirect bilirubin was in the control G1 7 days, 0.46±0.22 mg/dl; G2 laser 7 days, 0.75±0.43 mg/dl; G3 control 14 days, 0.54±0.37 mg/dl; G4 laser 14 days, 0.48±0.27 mg/dl. Statistical analysis of direct, total and indirect bilirubin did not present a significant difference when compared to the four groups.

Values=mean standard deviation; one-way ANOVA and the Tukey post-test; * p<0.05

**FIGURE 4:**
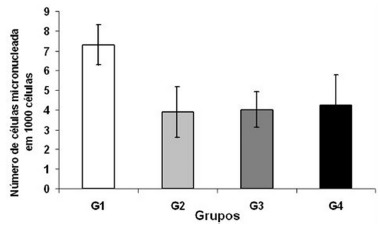
Number of micronucleated cells in 1000 peripheral blood erythrocytes analyzed between groups

Figure 4 shows the comparison of the number of micronuclei in peripheral blood erythrocytes among the different groups: control G1, 7,3±1 U; G2 laser 7 days, 3.9±1.3 U; G3 control 14 days, 4±0.9 U; G4 laser 14 days, 4.25±1.55 U. Statistical analysis did not present significant difference when comparing the four groups.

Values=mean standard deviation; one-way ANOVA and the Tukey post-test; * p<0.05

## DISCUSSION

In the comparison of liver weight between the groups, results were found that showed that the 14 day groups of the experiment, G3 and G4, were significantly higher than in the groups analyzed in 7 days, G1 and G2. However, there was no significant difference in the comparison of the two groups of 14 days with those of seven, as demonstrated by Aguiar et al^1^. Due to these results, there were no differences between the groups treated with laser of their respective control, and that the use of the laser in the remaining hepatic tissue does not cause delay in the restoration of the initial volume after the stimulus offered by HP.

In the morphological analysis for the number of hepatocytes there were no differences between the groups, without massive loss of hepatocytes, as already observed by other authors^17^ ^,^ ^21^.

In the analysis of the deposition of collagen fibers, its gradual reduction was observed, following the regeneration process of the remaining liver, in agreement with other studies where the TLBP does not add to the degradation of fibrotic areas, avoiding a posterior continuity of the cellular disarrangement^17, 21^.

The evaluation of liver function is based on liver function tests through sensitive and specific markers, classified as hepatocellular activity; they are useful for hepatic diagnosis, prognosis and monitoring. The ALT and AST aminotransferases in the presence of hepatocyte membrane damage are released in large amounts into the bloodstream, observing elevated levels of ALT and AST^8^ ^,^ ^19^. The studies performed by Araujo Lima et al^2^ and Monteiro^17^, both with hepatectomized cirrhotic Wistar rats, demonstrated that liver function tests remain within normal limits. In this study, all groups presented mean AST values, lower than the reference values ​​in the literature (39-92 U/l)^27^, but were not statistically significant when compared to each other. There was a decrease in ALT in the 14-day control group, already mentioned by other authors^20^.

The enzyme FA is distributed in several tissues; in the liver, is found in the canalicular membrane of the bile ducts. Impaired normal bile flow leads to cholestasis^14^. Araujo Lima et al.^2^, when analyzing their behavior after 30% hepatectomy (left lobe withdrawal) from cirrhotic rats, concluded that in all cirrhotic groups FA values ​​were higher than control, but there were no differences between the cirrhotic groups. In this study, no significant statistical differences were found between groups in their concentration; however, there was a decline in plasma levels in the two groups irradiated with laser when compared to the control, and it was deduced that the application of laser on the remaining hepatic tissue reduces the levels of AF, discharacterizing lesion in the canalicular membrane of the bile ducts.

GGT is present in several organs. In the liver, it is located in the canaliculi of hepatic cells and in the epithelial cells lining the bile ducts^18^. Its elevation in plasma occurs when there is cholangiocyte lesion^14^. This study showed that TLBP did not produce significant effects on plasma levels between groups. However, GGT is a nonspecific marker of liver disease and may be useful in determining whether or not an increased level of AF is due to liver disease or not^18^.

The high plasma concentration of bilirubin suggests the presence of hepatic lesions, due to the impediment or reduction of bile flow, where it is difficult to conjugate and reflux conjugated bilirubin to the blood^14^. Its analysis is useful for the differential diagnosis of liver and biliary diseases. The high concentration in plasma levels correlates with worse prognosis in hepatic insufficiency^8^. Aguiar et al.^1^ when evaluating the effect of hepatic regeneration after HP in congestive livers due to the induction of portal hypertension through the measurement of plasma levels of direct and indirect total bilirubin, did not observe a significant difference in their dosage. Similar results were observed in this study in which serum bilirubin levels remained within normal limits.

The presence of micronucleations in erythrocytes of bone marrow or peripheral blood erythrocytes suggests spontaneous genetic alterations or induction by means of genotoxic agents^5^. In this study we opted for the use of the micronucleus test in peripheral blood erythrocytes. This choice was due to the fact that it is a simple method, which has good reproducibility, rapid analysis, and requires only small amounts of peripheral blood, besides being less invasive^9^. This test yielded results similar to those of Monteiro^17^, without statistical differences between the different groups, in the count of micronucleations in peripheral blood erythrocytes, demonstrating values ​​not consistent with the presence of nuclear damage.

## CONCLUSION

The use of low-power laser in the dose applied on the remaining hepatic tissue after partial hepatectomy does not interfere with normal hepatic function and does not lead to mutagenic damage.
